# Protective Effect of Ginsenoside CK against Autoimmune Hepatitis Induced by Concanavalin A

**DOI:** 10.3390/foods12244379

**Published:** 2023-12-05

**Authors:** Jingjing Zhang, Yao Liu, Chao An, Chen Liu, Saijian Ma, Qiwen Zhang, Hao Ding, Jingjing Shao, Wenjiao Xue

**Affiliations:** Shaanxi Institute of Microbiology, Shaanxi Key Laboratory of Qinling Ecological Security, Xiying Road 76, Xi’an 710043, China; zhangjj@xab.ac.cn (J.Z.); liuyao@xab.ac.cn (Y.L.); anchao@xab.ac.cn (C.A.); liuch@xab.ac.cn (C.L.); masj@xab.ac.cn (S.M.); zhangqw@xab.ac.cn (Q.Z.); dinghao@xab.ac.cn (H.D.); shaojj@xab.ac.cn (J.S.)

**Keywords:** autoimmune hepatitis, ginsenoside CK, T cells, TLR4

## Abstract

Ginsenoside CK, a kind of rare ginsenoside transformed from protopanaxadiol saponins extracted from the genus Panax, has been proven to possess favorable bioactivities such as anti-inflammatory, anti-cancer, anti-diabetes, and hepatoprotective effects. The current study is targeted to determine the effect of ginsenoside CK on hepatitis induced by concanavalin A (Con A). Mice were treated with different dosages of ginsenoside CK for 7 days, and Con A (15 mg/kg) was intravenously injected to induce autoimmune hepatitis (AIH) after the last administration. The results demonstrated that pretreatment with ginsenoside CK (40 mg/kg) could obviously ameliorate the increase in serum indicators related to liver function such as AST, ALT, and ALP, and hepatic lesions induced by Con A. Meanwhile, ginsenoside CK suppressed hepatocyte apoptosis, which was observed in pathological data, and immunoblotting results showed that the expression of Bax, Bcl-2, and other proteins was regulated by CK. Furthermore, the release of inflammatory cytokines such as tumor necrosis factor-α (TNF-α) and IL-6 in mice with AIH were lowered by the administration of 40 mg/kg of ginsenoside CK. Furthermore, ginsenoside CK elevated the gene expression of Nrf2 and Sirt1 and augmented downstream target genes such as HO-1. In addition, a significant inhibition effect of the TLR4/NF-κB signal was observed in 40 mg/kg CK-pretreated mice compared with the model group. To sum up, the results indicated that ginsenoside CK has a notable hepatoprotective effect against AIH by activating Sirt1/Nrf2 and suppressing the TLR4/NF-κB signaling pathway.

## 1. Introduction

The liver, working as an essential digestive organ, takes charge of the metabolism of multiple nutrition intake, which makes it the most active place for various chemical reactions. Above all, the liver is the major organ for drug biotransformation. Enzymes existing in the endoplasmic reticulum in liver cells could attach to the lipid layers of membranes to catalyze the metabolism of drugs into more water-soluble compounds [1]. However, the incidence rate of liver injuries has increased in recent years because of drug abuse, alcohol, viral infections, and so on. Autoimmune hepatitis (AIH) is a severe immune-mediated liver disease with unknown etiology and pathogenesis that can occur in children and adults of all ages [2]. Without appropriate medical treatment, AIH may develop into cirrhosis or even hepatocellular carcinoma (HCC). The clinical symptoms of AIH are strikingly elevated levels of serum alanine (ALT) or aspartate (AST) aminotransferases, the infiltration of abnormal abundant inflammatory cells in the liver, and hepatic necrosis [3,4]. The combination of prednisone or prednisolone with azathioprine is the main remedy for AIH with a favorable therapeutic effect. However, the side effects that accompany this therapy can occur with high probability, such as weight gain, emotional instability, and osteoporosis, which highlights the need to develop more safe and effective therapies for AIH.

Concanavalin A (Con A), a plant lectin with mannose/glucose-binding activity, is extracted from jack bean, which could play an effective role in the induction of liver inflammation and an immune reaction when administered intravenously or intraperitoneally and, therefore, has been chosen as an efficient chemical regent to induce the AIH model in rodents [5]. The mechanism of Con A-stimulated hepatic injury could be attributed to the increase in active CD4^+^ T cells and Kupffer cells and the subsequent expression of varied inflammatory indicators such as tumor necrosis factor-alpha (TNF-α) and interleukins (ILs) [6]. According to previous reports, several signaling pathways also participated in the hepatotoxic reaction of Con A. The NF-κB signaling pathway plays a critical role in the development of immune-related diseases because of its regulation effects on transcription factors, inflammatory cytokine production, and even cell survival. Toll-like receptors (TLRs) are an evolutionarily conserved group of pattern-recognition receptors that can recognize metabolites existing in pathogens [7]. The activation of TLRs could trigger the activation of NF-κB to initiate the immune response [7]. Therefore, targeting the TLR4/NF-κB pathway could be an effective therapeutic strategy for AIH.

Recently, increasing interest has been focused on natural products because of their favorable clinically therapeutic effects and fewer side effects. Various phytochemicals extracted from natural products have been explored with multiple biological activities such as antioxidation, anti-inflammation, and so on. Herein, we mainly focused on ginseng, a widely used Chinese traditional herbal medicine that contains many active ingredients such as saponins, polysaccharides, polypeptides, and volatile oils. Ginsenosides, which are triterpene saponins, have been reported as the signature bioactive ingredient of ginseng. During the past few decades, the therapeutic effects of various ginsenosides, especially rare ginsenosides, have been studied in both experimental and clinical research, including the anticancer effect and effects on improving immunity, diabetes mellitus, neuropathic disease, and cardiovascular diseases [8]. For instance, rare ginsenosides Rg3 and Rh2, which were transformed through deglycosylation directly from Rb1, Rb2, Rb3, Rc, and Rd, which are abundant in the crude extract of ginseng, have pharmacological effects on multiple symptoms by modulating various signaling pathways [9,10]. At present, these two ginsenosides have been proven by the State Drug Administration of China as drugs or health products that can be applied for adjuvant treatment of cancer [11]. Ginsenoside Compound K (CK) is another secondary ginsenoside produced by the biotransformation of major saponins, and the main metabolite exists in tissue or blood after oral administration of PPD ginsenosides [12]. Researchers monitored the pharmacokinetics of ginsenoside CK in rodents and humans. The results showed that the maximum concentration of ginsenoside CK in the blood of mice could reach 652 ng/mL after administration of 50 mg/kg, while the value in the human body could reach 733.9 ng/mL after oral administration of 200 mg/kg. The times of maximum concentration are 2.6 h and 3.3 h in mice and adults, respectively [12]. Additionally, studies have reported the favorable hepatoprotective effect of ginsenoside CK due to its beneficial bioactivities. For example, researchers indicated that ginsenoside CK could act as an antioxidant to counteract the hepatotoxicity induced by SVP and regulate iron homeostasis in vivo [13]. In vitro, CK also could protect hepG2 cells from the cytotoxicity caused by tert-butyl hydroperoxide [14]. However, the effect of ginsenoside CK on Con A-induced AIH has not been investigated yet. The current study aims to track the potential curative effects and underlying mechanisms of CK in an immune liver injury by establishing a hepatitis mice model with Con A injection.

## 2. Materials and Methods

### 2.1. Chemicals and Reagents

**Figure 1:**
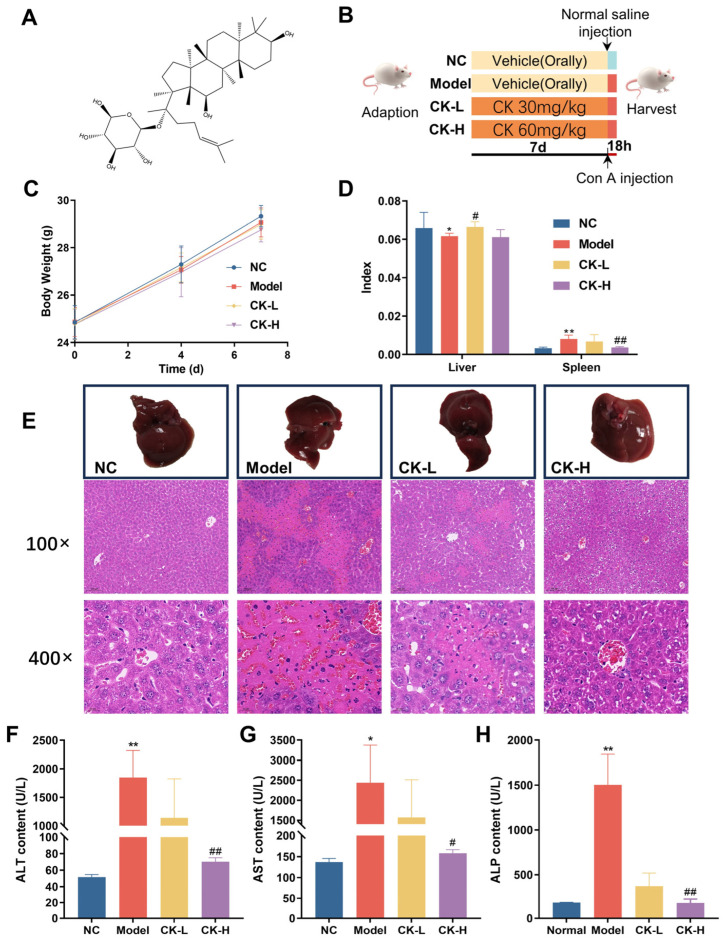
Ginsenoside CK ameliorates the liver injury caused by Con A injection. (**A**) Molecular structure of ginsenoside CK; (**B**) process of animal study; (**C**) effect of ginsenoside CK on the mice body weight before Con A induction; (**D**) organ index of each group; (**E**) morphology and H&E staining of liver tissue in each group; images with magnification ×100 and ×400 were recorded. Normal control group shows a normal arrangement of hepatocyte plates while Con A group shows obvious necrosis in liver tissues. CK 20 mg/kg and 40 mg/kg groups showed less necrosis. Effects of CK on the levels of alanine aminotransferase (**F**), aspartate aminotransferase (**G**), and alkaline phosphatase (**H**). * *p* < 0.05, ** *p* < 0.01 compared to normal group; # *p* < 0.05, ## *p* < 0.01 compared with model group.

Ginsenoside CK (purity ≥ 98.0%) was purchased from Sichuan Purify Biotechnology (Chengdu, China) (Figure 1A). Alanine aminotransferase (ALT), aspartate aminotransferase (AST), and alkaline phosphatase (ALP) detection kits were purchased from Rayto Life Sciences Co., Ltd. (Shenzhen, China). Superoxide dismutase (SOD), glutathione (GSH), and malondialdehyde (MDA) commercial kits were purchased from Nanjing Jiancheng Bioengineering Institute (Nanjing, China). The BCA protein concentration assay kit was obtained from Solarbio (Beijing, China). The RIPA lysis buffer was provided by Beyotime Biotechmology (Shanghai, China). Interleukin 6 (IL-6), Interleukin 1β (IL-1β), and tumor necrosis factor α (TNF-α) ELISA kits were purchased from Shanghai Enzyme-linked Biotechnology Co., Ltd. (Shanghai, China). Antibodies against F4/80, CD4, GAPDH, NF-κB, Bcl-2, and Bax were purchased from Proteintech (Rosemont, IL, USA). The p-IκBα antibody was obtained from Cell Signaling Technology (Danvers, MA, USA). Cleaved-caspase-3, cleaved-caspase 9, and the secondary antibodies were purchased from Abbkine Scientific Co., Ltd. (Wuhan, China). All other reagents were of analytical grade (Sinopharm Chemical Reagent, Shanghai, China).

### 2.2. Animals

Twenty male Kunming mice (6–8 weeks, body weight 20–22 g), purchased from Gempharmatech Co., Ltd. (Nanjing, China), were kept in polypropylene cages and acclimated under controlled and pathogen-free conditions (12 h light/12 h dark cycle at a temperature of 22 ± 2 °C and a humidity of 50 ± 5%) with free access to laboratory-standard food and drinking water for one week. The whole study protocol was conducted in accordance with the Animal Ethics Procedures and Guidelines of the People’s Republic of China and approved by the Northwest University Animal Ethics Committee (NWU-AWC-20210303M).

### 2.3. Experimental Design

After acclimation, mice were assigned to four groups according to their body weight with five mice per group: (1) normal control; (2) ConA alone; (3) ginsenoside CK at 20 mg/kg (CK low dose, CK-L) + ConA; and (4) ginsenoside CK at 40 mg/kg (CK high dose, CK-H) + ConA. The experimental process refers to the methods reported in the literature with some modifications [15]. The dosage of ConA was 15 mg/kg. For the normal control and ConA-alone groups, mice were treated with a vehicle (2% PEG400 dissolved in normal saline) orally every day. Mice in the CK-L and CK-H groups were intravenously administrated ginsenoside CK once daily for seven days. One hour after the last gavage, all mice except the vehicle control were injected with Con A through the tail vein while an equal volume of normal saline was given to the control group intravenously. Mice were sacrificed by cervical dislocation after they were challenged with Con A for 18 h. Liver, spleen, and blood samples were collected. Serum samples were obtained by centrifuging the blood and storing it at −80 °C for further detection. After weighing the liver and spleen tissues, they were separated into two parts: One was preserved at −80 °C for additional analysis and the other part was fixed in formalin for pathologic analysis (Figure 1B).

### 2.4. Histopathological Examination of Liver and Spleen

Tissues fixed with formalin for 24 h were then embedded in paraffin and cut into 5-μm sections, which were further analyzed by hematoxylin and eosin (H&E) staining. Antibodies of F4/80 (1:200), CD4 (1:750), Ki-67 (1:2000), TNF-α (1:500), and IL-6 (1:1000) underwent immunofluorescent or immunohistochemical staining [16]. In brief, sections were dewaxed and rehydrated followed by antigenic retrieval. After that, sections were treated with 3% hydrogen peroxide and 5% bovine serum albumin and incubated with the above primary antibodies overnight at 4 °C. For IF, sections were subsequently incubated with the FITC-conjugated secondary antibody at room temperature and DAPI was used for nucleus staining. For IHC, sections were treated with biotin-conjugated secondary antibodies at room temperature and stained with DAB. Nuclei were marked with hematoxylin. The slides were observed using fluorescence microscopy (Nikon, Japan) and recorded. Image J was used for the semi-quantitative analysis of CD4^+^ T cells’ infiltration.

### 2.5. Biochemical Analysis

Serum samples were used for the detection of hepatoxicity indices, including ALT, AST, and ALP using the corresponding kits according to the instructions of the manufacturer.

### 2.6. Quantitative Real-Time PCR

After grinding the liver tissues under liquid nitrogen, the total RNA was extracted using the Trizol reagent and analyzed by an Ultramicro-ultraviolet spectrophotometer (Nanodrop One, Thermo Scientific, Waltham, MA, USA), followed by reverse transcription-PCR using a RevertAid kit (Thermo Fisher, Waltham, MA, USA) according to the instructions. The obtained cDNA was mixed with the SYBR Green PCR mix (Roche, Basel, Switzerland) and qRT-PCR was performed on the Bio-rad CFX96 Touch (Bio-rad, Hercules, CA, USA). The experiment continued according to the following cycling conditions: initial step for Taq polymerase activation (a hot start at 95 °C for 5 min) then two-step cycling (44 cycles consisting of 10 s at 95 °C for DNA denaturation and 10 s at 60 °C combined annealing/extension). The primer sequences used are listed in Table S1. β-actin was used as a housekeeping gene. The relative expression of mRNA was estimated according to the 2^−ΔΔCt^ method.

### 2.7. Immunoblotting Analysis

The total protein of hepatic tissues was extracted using standard procedures [17]. Briefly, tissues were ground and lysed using RIPA lysis containing 1% PMSF for 20 min. After centrifugation, the supernatant was mixed with the loading buffer and heated for protein denaturation. Samples were separated by SDS-PAGE (12% gel) and transferred to a PVDF membrane, which was then blocked with skim milk, followed by incubation with the corresponding primary antibodies (Bax (1:2000), Bcl-2 (1:1000), c-caspase 3 (1:1000), c-caspase 9 (1:1000), p-IκBα (1:1000), and NF-κB p65 (1:2000)) overnight at 4 °C and peroxidase-conjugated secondary antibodies for another 1 h at room temperature. Images were captured using an Amersham Imager 600 (General Electric Company, Boston, MA, USA) and data were collected by ImageJ software (v1.8.0).

### 2.8. Inflammatory Markers Assessment

Expression levels of inflammatory indices such as TNF-α, IL-6, and IL-1β in liver tissue were detected utilizing ELISA kits according to the manufacturer’s instructions. In brief, the liver tissue was homogenized in normal saline followed by centrifuging at 4 °C for 15 min to separate the supernatant. The total protein was analyzed using a BCA kit for data normalization.

### 2.9. Statistical Analysis

Data are expressed as mean ± SEM. All data were analyzed using one-way ANOVA and the LSD test for multiple comparisons in SPSS (v24.0) or *t*-tests in Excel (v4.3.4.24). A *p*-value less than 0.05 was considered a significant difference.

## 3. Results

### 3.1. Ginsenoside CK Ameliorated Hepatic Injury Induced by Con A

During the first 7 days following the administration of ginsenoside CK, the body weight of mice in each group showed no remarkable differences, which demonstrated that ginsenoside CK has no influence on the body weight of mice (Figure 1C). After the injection of Con A, body weight decreased and the liver index showed a slight reduction in the model group compared to the normal control. However, improvements in the depressed body weight and liver index appeared in mice pretreated with ginsenoside CK, primarily indicating that CK may protect mice against the liver injury caused by Con A (Table S2, Figure 1D). The results of H&E staining revealed that Con A injection contributed to severe hepatic injury as extensive necrosis was observed (Figure 1E). The histopathological deterioration in mice treated with CK, in contrast, was significantly alleviated according to the reduced necrotic area and relatively regular hepatocyte arrangement.

To further assess the protective effect of CK on Con A-induced autoimmune hepatitis, levels of related enzymes including ALT, AST, and ALP in serum were evaluated. As shown in Figure 1F–H, markedly elevated contents of the serum indices in the model group emerged compared to those of the untreated control group. Interestingly, mice with CK pretreatment exhibited attenuated levels of the three enzymes when compared to those challenged with Con A alone.

Notably, these findings indicated the prevention effect of ginsenoside CK against acute liver injury induced by Con A in mice.

### 3.2. Ginsenoside CK Pretreatment Inhibited Cell Apoptosis in Mice with Hepatitis

**Figure 2:**
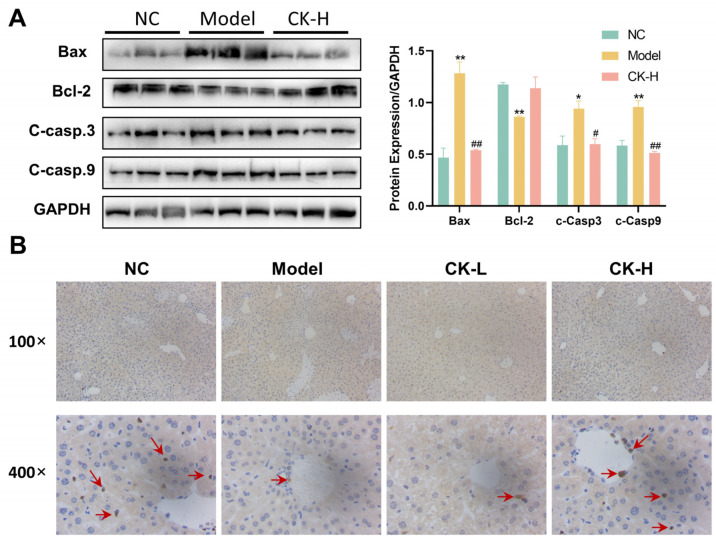
Effects of ginsenoside CK on cell apoptosis in mice with AIH. (**A**) Protein expression related to cell apoptosis in three groups; (**B**) immunohistochemical staining of Ki67 in liver tissues. Positive staining is indicated by a red arrow. * *p* < 0.05, ** *p* < 0.01 compared to normal group; # *p* < 0.05, ## *p* < 0.01 compared with model group.

After verifying the effect of ginsenoside CK on hepatic necrosis and the disruption of enzymes caused by Con A, the features related to hepatocellular apoptosis were explored by Western blot and immunohistochemical staining. As shown in Figure 2A, the injection of Con A substantially elevated the expression level of activated caspase-3 and caspase-9, as well as the level of Bax. Meanwhile, the expression of Bcl-2 decreased in liver tissue treated with Con A compared with the normal group, whereas these protein levels in mice were reversed under CK pretreatment. Moreover, immunohistochemical staining indicated that the positive expression of Ki67, a nuclear antigen that is closely related to cell proliferation, in the liver tissue of mice treated with Con A alone was obviously inhibited relative to the normal control, while upregulation emerged in the CK-L and CK-H groups (Figure 2B). These results supported the idea that ginsenoside CK could counteract hepatocellular apoptosis and facilitate cell proliferation in mice.

### 3.3. Ginsenoside CK Regulated Immune Imbalance Caused by Con A

**Figure 3:**
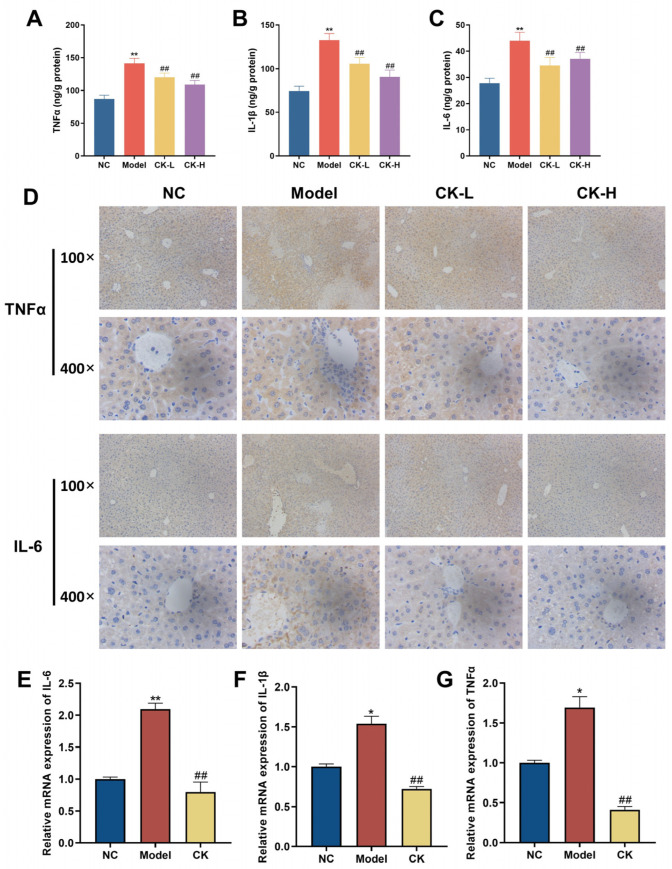
Ginsenoside CK regulates the expression of inflammation cytokines in liver tissue. Levels of TNFα (**A**), IL-6 (**B**), and IL-1β (**C**) in liver tissues were tested by ELISA; (**D**) immunohistochemical staining of TNFα and IL-6 of liver tissue in each group; gene expression of IL-6 (**E**), IL-1β (**F**), and TNFα (**G**) in normal control group, model group, and CK-H group was tested using qRT-PCR. Data are presented as mean ± SEM, * *p* < 0.05, ** *p* < 0.01 compared to normal group; ## *p* < 0.01 compared with model group.

**Figure 4:**
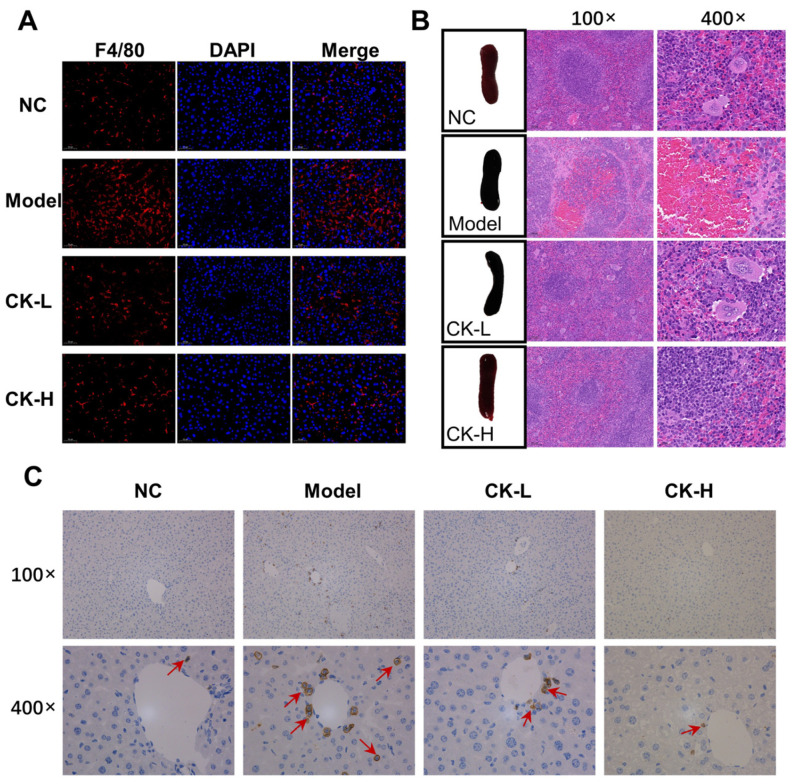
Ginsenoside CK improves the inflammation cell infiltration in liver tissue of AIH mice. (**A**) immunofluorescent staining of F4/80 in liver tissue of the four groups; F4/80 are marked with red fluorescence and cell nuclei are indicated with DAPI (blue); (**B**) morphology and H&E staining of spleen in each group. The regular structures shown in normal mice were seriously damaged in AIH mice, while mice with CK 20 mg/kg and 40 mg/kg showed an obvious recovery; (**C**) immunohistochemical staining of CD4 in liver. Positive staining is indicated by a red arrow. Con A group exhibited a significant increase in CD4^+^ cells compared to the normal control group, while CK-L and CK-H groups showed much lower positive staining of CD4^+^.

The inflammatory response plays an essential role in maintaining liver homeostasis; therefore, the levels of inflammatory factors such as IL-1β, IL-6, and TNF-α were first examined by ELISA. The data revealed that the contents of TNF-α, IL-6, and IL-1β in mice with Con A induction were elevated to 134.46 ± 23.5, 42.73 ± 9.82, and 126.45 ± 23.12 ng/g protein, respectively, which is nearly 1.5 times higher than that in the normal control group (87.01 ± 18.24, 27.77 ± 6.02 and 74.33 ± 17.36 ng/g protein). In contrast, data for the CK-L and CK-H groups showed that the levels dropped to different extents. The levels of TNF-α, IL-6, and IL-1β in the CK-H group reached 112.89 ± 15.07, 35.32 ± 5.33, and 93.02 ± 21.03 ng/g protein, respectively (Figure 3A–C). These results were further confirmed by immunohistochemical staining and RT-qPCR, as shown in Figure 3D,E. The data described that the gene expression of those indices, which markedly increased in the mice treated with Con A alone compared to the mice in the normal group, recovered to a lower state in the CK (40 mg/kg)-treated group. At the same time, the infiltration of inflammatory F4/80-positive macrophages, which significantly accumulated in the Con A group, was markedly reduced by CK pretreatment (Figure 4A).

Additionally, T cells have been revealed as the cardinal cells in the immunoregulation of acute immune hepatitis, so we compared the morphology of the spleen, which acted as the key immune organ, in the four groups. As shown in Figure 4B, the spleen in the AIH mice induced with Con A exhibited an abnormal dark red color and swelling, while the spleen of the healthy mice was bright red. Also, the weight of the spleen after Con A was noticeably increased compared with that in normal mice. However, mice with CK treatment displayed a palliative swelling degree and lower weight in spleen tissue, as listed in Table S2. Based on these results, IHC staining was conducted to detect the degree of CD4^+^ T cells’ infiltration in the liver. As presented in Figure 4C and Figure S1, injection of Con A significantly promoted the infiltration of CD4^+^ T cells into the hepatic tissue compared with that in the normal group, while CK pretreatment prevented the infiltration to some extent compared to the Con A group.

In brief, these data demonstrated that the pretreatment of ginsenoside CK was favorable for modulating immune imbalance and inhibiting inflammation in AIH mice.

### 3.4. Effects of Ginsenoside CK on Oxidative Stress Caused by Con A

**Figure 5:**
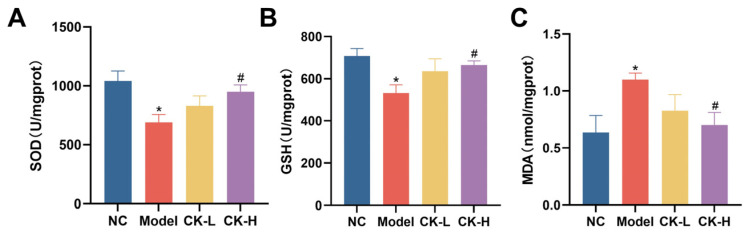
Ginsenoside CK neutralized the oxidative stress induced by Con A. Activity of superoxide dismutase (SOD) (**A**), reduced glutathione (GSH) (**B**), and the content of malondialdehyde (MDA) (**C**) were determined. Data are presented as the mean ± SEM.* *p* ˂ 0.05 vs. normal control group; # *p* ˂ 0.05 vs. model group.

Accompanied by the inflammatory response, peroxidation is closely involved with Con A-caused hepatitis. Thus, correlated indices such as SOD, GSH, and MDA were detected to evaluate the oxidative burden in the liver tissue of each group. As shown in Figure 5A–C, in comparison to the normal group, Con A apparently aggravated the oxidative damage in hepatic tissue as the contents of antioxidants including SOD and GSH were dramatically depressed while the level of MDA, a product formed by the breakdown of lipid peroxides, distinctly increased. Nevertheless, ginsenoside CK pretreatment moderated the lesions in peroxidative markers and improved the activity of antioxidants in liver tissues compared to that in the model group.

### 3.5. Effects of Ginsenoside CK on Sirt1/Nrf2 Signaling Pathway in the Liver

**Figure 6:**
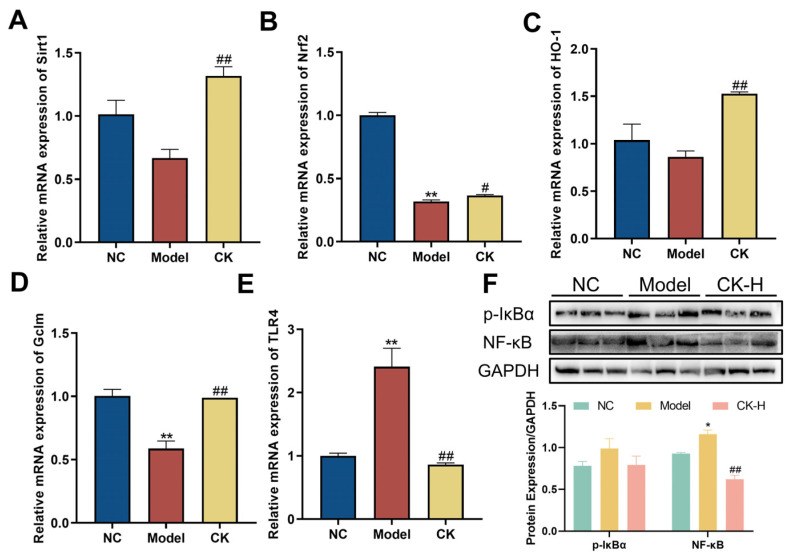
Ginsenoside CK regulated Sirt1/Nrf2 and TLR4/NF-κB pathways. mRNA levels of Sirt1 (**A**), Nrf2 (**B**), HO-1 (**C**), Gclm (**D**), and TLR4 (**E**) in liver tissues of normal control group, model group, and CK-H group were detected by RT-qPCR; (**F**) Western blotting showed the protein expression of p-IκBα and NF-κB. Data were expressed as mean ± SEM. * *p* < 0.05, ** *p* < 0.01 vs. normal control group, # *p* < 0.05, ## *p* < 0.01 vs. model group.

In order to explore the influence of CK on the Sirt1/Nrf2 signaling pathway, the total RNA of hepatic tissue was extracted to detect the relative gene expression. According to the data presented in Figure 6A,B, stimulation of Con A could dramatically downregulate the mRNA expression of Sirt1 and Nrf2 in the liver compared to that of mice without interference. In addition, the levels of downstream genes such as HO-1 and GCLm were also reduced when exposed to Con A intervention (Figure 6C,D). Interestingly, the administration of ginsenoside CK obviously enhanced the gene expression of the genes mentioned above, which indicated the modulative effect of ginsenoside CK on the Sirt1/Nrf2 signaling pathway.

### 3.6. Ginsenoside CK Targeted TLR4/NF-κB Signaling Pathway to Safeguard Hepatocytes from Damaged by Con A

NF-kB is closely related to multiple physiological processes, especially cell apoptosis and inflammatory response. The expression of the NF-κB protein was identified by Western blot and RT-qPCR to determine the effects of ginsenoside CK on this signaling pathway. The results of Western blotting showed that the expression of p-IκBα and NF-κB p65 in the liver tissue of the Con A-treated group was significantly higher than that in the normal group. However, pretreatment with ginsenoside CK notably downregulated the level of p-IκBα compared to the model group, as well as the expression of NF-κB p65 (Figure 6F). Moreover, the gene level of TLR4, which could activate the cascade of NF-κB signaling, was examined by RT-qPCR. The data illustrated that mice induced with Con A exhibited a rising trend in the mRNA level of TLR4 in the liver, whereas ginsenoside CK reduced the expression of TLR4 (Figure 6E). Above all, the regulation of these modulators demonstrated that CK might protect the liver from autoimmune injury caused by Con A by blocking the activation of the TLR4/NF-κB signaling pathway.

## 4. Discussion

**Figure 7:**
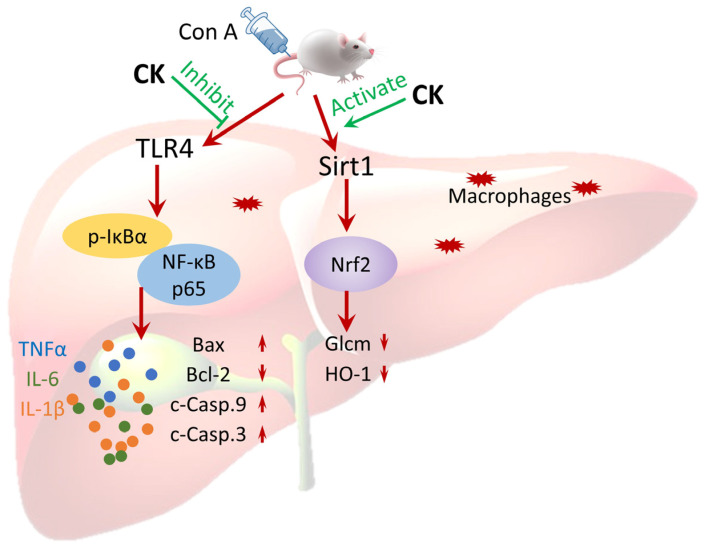
Possible mechanisms of hepatoprotective activity of ginsenoside CK against autoimmune hepatitis induced by Con A.

Ginsenoside CK, absent naturally, is a protopanaxadiol (PPD)-type ginsenoside generated from other major ginsenosides by intestinal microflora metabolism and endowed with remarkable pharmaceutical activities, including anticancer, immunoregulation, and improved effects on metabolic diseases and neurodegenerative diseases. Previous studies have proved that ginsenoside CK could block the cell cycle of Hela cells in the G0/G1 phase at 60 μM and inhibit cell metastasis [18]. Another study revealed that ginsenoside CK at 40 mg/kg and 80 mg/kg could significantly inhibit tumor growth in the mice triple-negative breast cancer (TNBC) model by modulating glutamine metabolism. The tumor weight inhibition rates reached 32.42% (40 mg/kg) and 56.51% (80 mg/kg), respectively [19]. Furthermore, 10 mg/kg ginsenoside CK could also alleviate hyperglycemia and insulin resistance in db/db mice by regulating skeletal muscle insulin sensitivity [20]. Our findings first investigated the protective effect of ginsenoside CK against Con A-induced AIH and the potent antioxidant, anti-inflammatory, and anti-apoptosis activity through modulating Sirt1/Nrf2 and TLR4/NF-κB signaling pathways (Figure 7).

Immunological liver injury, characterized by elevated inflammatory cells infiltrating in liver tissue and the boosted levels of transaminase and inflammatory cytokines, could trigger other severe liver diseases such as liver cirrhosis and hepatocarcinoma [21,22]. AIH is one of the immunological liver diseases that occurs globally [2]. Con A-induced mouse hepatitis has been successfully used to simulate human AIH and acute viral hepatitis because of its similar pathogenesis [16]. Con A administrated intravenously could bind strongly to mannose-rich glycoproteins existing in the liver sinusoidal., which initiate the activation of T cells, particularly the CD4^+^ T cells in the hepatic tissue [23]. The activated T cells stimulated other immune cells such as macrophages and the secretion of various cytokines such as TNF-α and ILs, which rapidly cause inflammatory immune-mediated liver injury, as shown by the drastic increase in the levels of serum ALT and AST, two signature cytosolic enzymes that will release into the blood while hepatocellular death occurs, and extensive hepatic necrosis [24]. In accordance with the previous studies, our research verified the injurious effects of Con A injection on liver tissue via biochemical tests and a histopathological assay where the results exhibited significant elevation in indices of hepatotoxicity and severe hepatocyte necrosis after treatment with Con A alone. In contrast, the present study indicated that pretreatment with ginsenoside CK can prevent liver injury by downregulating the expression of ALT, AST, and ALP, while also ameliorating liver necrosis in mice with AIH (Figure 1).

Furthermore, patients suffering from AIH showed severe inflammatory cell infiltrates in liver tissue, which could release various cytokines and chemokines to further aggravate the inflammatory injury [25]. In our study, Con A injection caused an apparent infiltration of F4/80 macrophages in the liver and significant elevation in the production of proinflammatory cytokine IL-6, TNF-α, and IL-1β, which is in line with earlier research, while ginsenoside CK administration could effectively reduce macrophage infiltration and the release of cytokines (Figure 3 and Figure 4). Moreover, CD4^+^ T cells are important immune cells in the human immune system, which directly reflect the immune function of the human body. Scholars have highlighted the pivotal role of CD4^+^ T cells in the development of AIH using different mouse models [26]. The transfer of CD4^+^ T cells extracted from mice with AIH could induce liver inflammation in recipient mice [26]. According to our research, CD4^+^ T cells’ infiltration into the liver was aggravated as the volume of the spleen was enlarged after treatment with Con A. Along with the recovery of the liver injury in the mice treated with ginsenoside CK, the morphology of the spleen improved and the number of CD4^+^ T cells was reduced as well. The results indicated that pretreatment with ginsenoside CK can prevent AIH in mice by modulating the immunoreaction.

The inflammatory response also could result in hepatocyte apoptosis, another vital factor in the occurrence and development of Con A-induced liver injury, and may contribute to the liver fibrosis and cirrhosis seen in AIH patients [27]. During cellular apoptosis, Bax (Bcl-2-associated X protein), a symptomatic proapoptotic gene, will execute a molecular conformational change and translocation into the mitochondrial outer membrane to initiate the Caspase cascade, prompting consequent apoptosis in a caspase-dependent manner. Bax also could antagonize Bcl-2 and other anti-apoptotic proteins to neutralize their anti-apoptotic effects. Our research revealed that Con A significantly increased the protein expression of Bax and other downstream caspases such as cleaved-caspase9 and cleaved-caspase 3 accompanied by a decrease in the level of Bcl-2, which is in harmony with previous results reported by Li et al. who dictated that Con A caused cellular apoptosis in hepatic tissue [28]. In contrast, mice administrated ginsenoside CK showed a reduction in Bax and caspase protein levels and enhancement of Bcl2 expression. What is more, the expression of Ki-67, a cell proliferation marker, also revealed that ginsenoside CK protected hepatocytes against the Con A attack (Figure 2). Taken together, the data suggested that the amelioration effect of ginsenoside CK on hepatitis is closely related to its anti-apoptotic activity.

In addition to the inflammatory injury and immunologic derangement, Con A could also induce peroxidative damage in the liver by stimulating reactive oxygen species (ROS) generation, which was considered another main culprit in the pathogenesis of AIH [29,30]. Patients with AIH showed a higher number of ROS in livers than in healthy people [31]. ROS is a group of chemical species that is highly reactive and closely involved in cellular proliferation, differentiation, and death. Redundant ROS could attack the cellular membrane and mitochondria and damage cellular components such as lipids, saccharides, nucleic acids, and proteins, finally causing cell injury or apoptosis [32]. In addition, it has been reported that ROS can participate in the activation of multiple signaling pathways related to intracellular inflammation such as NF-κB, MAPK, and Keap1-Nrf2 [32]. In our research, the data proved that the liver tissue suffered peroxidative damage after Con A administration based on the upregulated MDA level, one of the peroxidation products of polyunsaturated fatty acids, and the reduction in SOD and GSH, two main indexes on behalf of the liver’s antioxidant capability. It is remarkable, however, that mice pretreated with ginsenoside CK presented with a relatively lower content of MDA and a significant increase in antioxidase activity (Figure 5). Therefore, these results validate that the hepatoprotective activity of ginsenoside CK may be partially attributed to its attenuation effect on oxidative stress.

Antioxidant defense systems are regulated by complex and diverse signaling pathways to strike a balance between promoting and suppressing oxidative stress. Among them, nuclear factor erythroid 2-related factor 2 (Nrf2), a transcription factor that plays a central role in various pathological conditions, is an emerging target for cellular resistance to oxidants. Once oxidation occurs, Nrf2 is activated by releasing kelch-like ECH associating protein 1 (Keap1), which is a negative regulator that forces Nrf2 into an inert state under normal conditions and transferred into the cell nucleus, concomitantly stimulating downstream antioxidant expression to defend against the cytotoxic effects of oxidative stress, including GCL, NQO1, and HO-1, several of the inducible enzymes involved in maintaining cellular homeostasis that can protect hepatocytes from oxidative stress-caused damage [33]. Previous research studies have reported that the knockout of Nrf2 in mice makes them more susceptible to the inflammatory liver injury induced by Con A [34]. Alternatively, plenty of reliable evidence indicated that the silent information regulator Sirtuin 1 (Sirt1), which has been regarded as a pivotal regulator in inflammation, apoptosis, and antioxidant defense systems, reduces ROS production by stimulating the Nrf2 pathway [35]. Studies have shown that Sirt1-deficient mice may develop an autoimmune-like disease, along with large accumulations of immune complexes in the liver and kidneys [36]. In this paper, Con A injection led to downregulation of the mRNA level of Sirt1, Nrf2, and the related downstream genes, which is consistent with previous studies. Conversely, ginsenoside CK pretreatment significantly enhanced the mRNA expression of these genes, which illustrated that the antioxidant effect of ginsenoside CK may account for the activity of modulating the Sirt1/Nrf2 pathway (Figure 6A–D).

The NF-κB (nuclear factor kappa-B) signaling pathway has been proven to be closely involved in multiple cellular activities including cell proliferation, inflammation, immune response, and so on. Researchers have demonstrated the activation of the NF-κB pathway and the elevated expression of downstream genes in mice injected with Con A [37]. Additionally, TLR is a highly conserved family comprised of innate immune cell receptors, which could recognize pathogen-associated molecular patterns and facilitate the expression of cytokines to modulate the innate and adaptive immune response. Among them, Toll-like receptor 4 (TLR4) has been proven to play a vital role in a variety of diseases such as cancers, cardiovascular diseases, and, especially, pathological conditions in the liver including liver inflammation, hepatic steatosis, and liver fibrosis. Ample evidence has verified that the activation of TLR4 could induce the expression and secretion of inflammatory factors by promoting the translocation of the p65 protein to initiate NF-κB signaling [38]. Moreover, previous research demonstrated that the suppression of TLR2/4 ligand release could alleviate liver damage caused by AIH. Our data confirmed that Con A activated the TLR4/NF-κB signaling pathway by upregulating the expression of TLR4 and the phosphorylation of IKBα, while the application of ginsenoside CK reversed this state, indicating that ginsenoside CK improved AIH in mice caused by Con A by regulating the TLR4/NF-κB signaling pathway (Figure 6E,F). However, the therapeutic effect of ginsenoside CK against acute liver injury has not been investigated. Previous studies have demonstrated that CK could act as a therapeutic agent for NAFLD by alleviating hepatic inflammation and peroxidation damage [39], which are also the main factors in AIH development. Based on that, we deduced that CK may also be a potential drug for the therapy of liver injury.

## 5. Conclusions

This study systematically presents the effects and pharmacological mechanisms of the rare ginsenoside CK on the prevention of AIH. Our research demonstrated that CK possesses a favorable hepatoprotective effect against AIH caused by Con A due to its admirable activity of regulating inflammation, oxidation, and cellular apoptosis. The underlying mechanisms of this effect are ascribed to the negative modulation of the TLR4/NF-κB and Sirt1/Nrf2 pathways. In summary, this study presents a theoretical basis for the clinical application of ginsenoside CK as an effective prophylactic agent for AIH.

## Data Availability

All data are contained within the article and the Supplementary Materials.

## Supplementary Materials

The following supporting information can be downloaded at: https://www.mdpi.com/article/10.3390/foods12244379/s1, Table S1: The primer sequences for qRT-PCR; Table S2: The absolute weight of body, liver, and spleen in each group. Data are shown as mean ± SEM. * *p* ˂ 0.05, ** *p* ˂ 0.01 vs. normal control group; # *p* ˂ 0.05, ## *p* ˂ 0.01 vs. model group; Figure S1: Ginsenoside CK decreases the CD4^+^ T cell infiltration in liver tissue of AIH mice. Data are shown as mean ± SEM. ** *p* ˂ 0.01 vs. normal control group; ## *p*˂ 0.01 vs. model group.
