# Supplementary material for: Protective Effect of Ginsenoside CK against Autoimmune Hepatitis Induced by Concanavalin A
Source: Foods. 2023 Dec 5;12(24):4379. doi: 10.3390/foods12244379 (PMC10743209; doi:10.3390/foods12244379)
Supplement: Supplementary file 1 [file foods-12-04379-s001.zip › foods-2730394-supplementary.pdf]

Table S1 The primer sequences for qRT-PCR.

Gene	Forward (5'-3')	Reverse (5'-3')
SIRT1	CGATGACAGAACGTCACACG	ATTGTTCGAGGATCGGTGCC
Nrf2	AAGAATAAAGTCGCCGCCCA	AGATACAAGGTGCTGAGCCG
GCLm	TAAGAAGGCGGCTTGATGCT	TGTGGTGAGTCCAACTGAGC
HO-1	CCTCACAGATGGCGTCACTT	TGGGGGCCAGTATTGCATTT
TNF-α	GCTGTTGCCCCTGGTTATCT	ATGGAGTAGACTTCGGGCCT
IL-6	AGTCCTTCCTACCCCAATTTCC	GGTCTTGGTCCTTAGCCACT
IL-1β	GCCACCTTTTGACAGTGATGAG	AGCTTCTCCACAGCCACAAT
TLR4	GCCATCATTATGAGTGCCAATT	AGGGATAAGAACGCTGAGAATT
β-actin	TGAGCTGCGTTTTACACCCT	GCCTTCACCGTTCCAGTTTT

Table S2 The absolute weight of body, liver and spleen in each group. Data are shown as mean ± SEM. **p* < 0.05, ***p* < 0.01 vs. normal control group; #*p* < 0.05, ##*p* < 0.01 vs. model group.

**Groups**	**Absolute Weight (g)**		
	**Body Weight**	**Liver**	**Spleen**
Normal	29.06±0.83	1.95±0.12	0.10±0.01
Model	24.32±0.78**	1.50±0.06*	0.20±0.02**
CK-L	24.33±0.58	1.61±0.03	0.16±0.05
CK-H	27.32±0.68$^{\#}$	1.67±0.08	0.10±0.01$^{\#\#}$

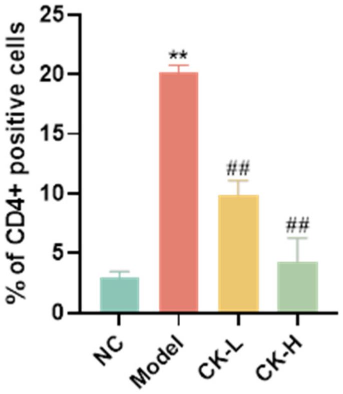

Figure S1 Ginsenoside CK decreases the CD4+ T cells infiltration in liver tissue of AIH mice. Data are shown as mean ± SEM. ***p* < 0.01 vs. normal control group; ##*p* < 0.01 vs. model group.
